# Diagnostic potential of serum HSP90 beta for HNSCC and its therapeutic prognosis after local hyperthermia therapy

**DOI:** 10.1371/journal.pone.0281919

**Published:** 2023-11-09

**Authors:** Neena G. Shetake, Amit Kumar, Nagraj Huilgol, Badri N. Pandey

**Affiliations:** 1 Radiation Biology & Health Sciences Division, Bhabha Atomic Research Centre, Mumbai, India; 2 Homi Bhabha National Institute, Mumbai, India; 3 Department of Radiation Oncology, Dr. Balabhai Nanavati Hospital, Mumbai, India; Uniwersytet Medyczny w Bialymstoku, POLAND

## Abstract

The present pilot study aims to investigate the diagnostic and prognostic efficacy of serum HSP90 beta in Head and Neck Squamous Cell Carcinoma (HNSCC) patients subjected to localized hyperthermia therapy (HT). Serum levels of HSP90 beta were measured by ELISA and its diagnostic and prognostic efficacy was determined by receiver operating characteristic curve (ROC) analysis. HNSCC patients showed significantly (P<0.05) higher serum levels of HSP90 beta (65.6±13.08 ng/ml) compared to Healthy Controls (HC: 23.5±3.8 ng/ml). No significant difference was observed in serum HSP90 beta levels between complete responders (CR) and non-responders (NR) in the chemo-radiation therapy (CRT) cohort. However, in CRT+HT cohort, CR showed significantly (P = 0.02) lower serum HSP90 beta levels at 24 h after HT (25.6±9.04 ng/ml) compared to NR (130.5±34.2 ng/ml). Youden’s index values between HNSCC versus HC, CR versus NR (CRT) and CR versus NR (CRT+HT) were found to be 0.47, 0.45 and 0.80, respectively. Thus, alterations in the serum HSP90 beta after HT suggest its potential in prognosis of HT response in HNSCC patients. Elevated levels of HSP90 beta may serve as a promising diagnostic serum bio-marker for HNSCC. However, further validation in larger patient samples is needed for clinical translation of HSP90 beta as diagnostic and prognostic biomarker.

## 1. Introduction

Head and neck cancer is the sixth most predominant cancer in the world and ~90% of them arise from squamous cell epithelium [1, 2]. In India, head and neck squamous cell carcinoma (HNSCC) is a major concern, as it covers one third amongst all cancers compared to 4–5% in the developed world [3, 4]. Moreover, 60 to 80% of patients in India present with advanced disease (stage III/IV) as compared to 40% in developed countries [5]. Currently, in India, HNSCC is predominantly managed by surgery, chemo-radiotherapy, targeted therapy and immuno-therapy [6]. Huilgol et al, demonstrated the significant potential of hyperthermia therapy (HT) to enhance the efficacy of chemo-radiation in HNSCC in terms of increasing overall survival, disease-free survival and quality of life without increasing the risk of complication or additional toxicities [7]. Thus, HT presents a promising approach for the treatment of HNSCC by improvement of clinical response and reducing the toxicities of radio- and chemo-therapy [8, 9].

In recent years, research has been aimed at developing targeted molecular therapeutics for the management of HNSCC [10–12]. However, despite the improvement in the treatment modalities and development of targeted molecular therapeutics, there has been only modest improvement in the overall survival of HNSCC patients [10]. This seems to be associated with the late diagnosis as well as the advanced clinical stage at the time of diagnosis. Thus, early diagnosis has become imperative for reduction in the current mortality rate in HNSCC patients. Several recent studies have examined plasma [13, 14], serum [15, 16], saliva [13] and tissue biopsy [17] samples to identify clinically significant bio-markers for HNSCC patients. Tissue bio-markers pose challenges owing to the heterogeneous nature of HNSCC involving different sites ranging from oral cavity to upper aero-digestive tract. Moreover, obtaining biopsy samples at different points of therapy is not feasible. On the contrary, serum or plasma based bio-markers are expected to have better diagnostic value due to ease of sample collection and better representation of tumor secretory profile.

Heat shock proteins (HSPs) have been known to be elevated in the serum/plasma samples of several cancer patients, especially solid tumors of lungs [18, 19] and liver [20]. HSPs are of particular interest due to their role in modulation of thermo-tolerance, which in turn affects the response of the tumors to HT. Tu et al, demonstrated the modulation of HSP70 and HSP90 expression in gastric tumors after transient hyperthermic intra-peritoneal chemoperfusion (HIPEC) treatment [21]. Their findings suggested that the application of second round of HIPEC after a gap of 24 h could minimize the chemo- and thermo-tolerance induced by elevated serum levels of HSP70/90 after first round of HIPEC. Amongst HSPs, HSP90 (particularly the alpha and beta isoforms of HSP90) have been implicated for the diagnostic potential in case of lung [18, 19] and liver cancer [20]. However, a definitive correlation of the two isoforms of HSP90 and their modulation in cancer patients has not yet been established. Besides, reports do not exist that study the level of HSP90 in HNSCC and its variation in response to HT. Our previous studies suggested the role of HSP90 in the mechanism of radio-sensitization and thermo-sensitization after magnetic hyperthermia therapy (MHT) in mouse fibrosarcoma tumor model [22, 23]. Thus, the aim of the present study is to validate the potential of HSP90 as diagnostic and prognostic marker in clinical scenario. For this serum samples of HNSCC patients were evaluated for the levels of HSP90. HNSCC cancer type was chosen as conventionally HNSCC patients are subjected to HT to improve the efficacy of chemo-radiotherapy (CRT), thereby making them a suitable model to evaluate the prognostic potential of HSP90 for predicting the response to HT.

## 2. Methods

### 2.1 Tumor details and treatment plan in HNSCC

During November 2016 to May 2019, total 15 HNSCC patients from Radiation Oncology Department, Dr Balabhai Nanavati Hospital, Mumbai were enrolled in this study with patients with curative intent. Necessary written consent from the patients and ethical approval from the Institute has been taken for the study. Healthy subject samples (age-matched) were obtained after appropriate ethical approval from BARC hospital. The patients with squamous cell carcinoma (SCC) comprised of the primary tumor sites of pyriform fossa (n = 4), tongue (n = 3), supra-glottis (n = 2), vocal cord (n = 1), uvula (n = 1), lung (n = 2), gingivobuccal sulcus (n = 1) and pharyngeal wall (n = 1). The SCC was confirmed by histopathology and the tumor staging status classified according to the eighth edition of American Joint Committee on Cancer (AJCC) classification [24], showed most of the tumors to be localized (T2-T4, N = 12). Three out of 15 tumors involved the lymph nodes with staging of T3N1, T4N2 and T3N2. Prior to HT, all the patients had been subjected to radiation therapy (average fractionated dose of 65 ± 5.2 Gy, each fraction of 2 Gy) and chemotherapy (cisplatin 60 mg/week for 6 doses). Radiation was delivered with conventional fractionation (2 Gy/day 10 Gy/week, total dose: 66–70 Gy) using Linear Accelerator with Intensity Modulated Radiation Therapy or 3 dimensional conformal therapy. Chemotherapy was infused once a week on any convenient day. Chemotherapy was not infused on the day of hyperthermia. Hyperthermia was delivered after pre-cooling for a period of 30–40 minutes, once every week ~1 h after the radiation therapy session. Hyperthermia always followed radiotherapy. HT was delivered to the patients (following a pre-cooling step) on Thermatron, a radiofrequency (RF) machine operating at 8.2 MHz. A pair of antennae was placed across the solid tumor, guided by visible tumor or anatomical landmarks. The power input was started after impedance matching input varied from 400 to 1000 kW. Power was gradually escalated unless the patients complained of unbearable pain, stress or discomfort. Power was then reduced and maintained till completion of the treatment. HT was stopped if the patient developed ≥ grade II thermal burns. Whenever feasible, the temperature attained during HT was measured by invasive thermometry with a thermistor probe. The average HT temperature to which the tumors were exposed was 41.87 ± 0.83°C. Patients received HT (1 to 9 weekly sessions) for 30 minutes after pre-cooling for 10 minutes. Patients’ response to the treatment was assessed at the end of the treatment cycle [7]. Response of patients to therapy was categorized according to RECIST (Response Evaluation Criteria in Solid Tumors) criteria. Accordingly, disappearance of all tumor lesion was categorized as complete response, reduction of >30% in tumor size as partial response (PR), <30% reduction in tumor size as stable disease (SD) and growth of >20% or occurrence of new lesions as progressive disease (PD) [25]. Patients with complete response were categorized as complete responders (CR), whereas, patients with PR, SD or PD were categorized as non-responders (NR) to treatment. The progression free survival ranged from 12 to 30 months with an average of 18.6 ± 3.41 months for CR as against 2.8 ± 0.91 months for NR (range: 1 to 6 months). Serum samples of healthy donors were collected after taking appropriate approval from BARC ethical committee.

### 2.2 Serum collection and storage

For ELISA analysis, 5 ml of blood was collected and serum was isolated by centrifugation (2200 RPM for 15 min) within 1 h of blood collection. Serum samples were collected at two time points, viz., before HT (but after completion of CRT) and 24 h after completion of HT session. The serum samples were stored at -80°C till further analysis. Repeated freeze-thaw cycles were avoided.

### 2.3 ELISA analysis

The levels of HSP90 beta were determined by using human specific ELISA kits (Cusabio, USA) following manufacturer’s instructions. Values lower than the minimum detectable dose (MDD) of the kit have not been considered and labeled as non-detectable (ND). Anonymized data for serum levels of HSP90 beta in HNSCC and HC is mentioned in (S1 and S2 Tables). In case of HNSCC patients, 1 out of 15 patients showed serum level of HSP90 lower than the MDD. Similarly, in case of HC, 10 out of 42 Samples showed non-detectable levels of HSP90 beta in serum. Therefore, for statistical analysis 14-HNSCC and 32-HC samples have been considered.

### 2.4 Statistical analysis

Statistical significance was determined by Mann-Whitney test and two sample t-test using Origin Pro 8.0 software. The paired comparison of ROC curves and determination of Youden index were performed using MedCalc Software ver 18.11.6 and web-based tool for ROC analysis (Epitools, http://epitools.ausvet.com.au). The optimum cut off value was determined by using the quantity corresponding to the maximum value of Youden’s index (Youden’s index = sensitivity+specificity-1).

### 2.5 Ethics approval

All procedures performed in studies involving human participants were in accordance with the ethical standards of the Dr. Balabhai Nanavati Hospital, Mumbai and with the 1964 Helsinki Declaration and its later amendments or comparable ethical standards.

## 3. Results

### 3.1 Serum levels of HSP90 beta were found to be significantly higher in HNSCC as compared to healthy controls (HC)

**Fig 1:**
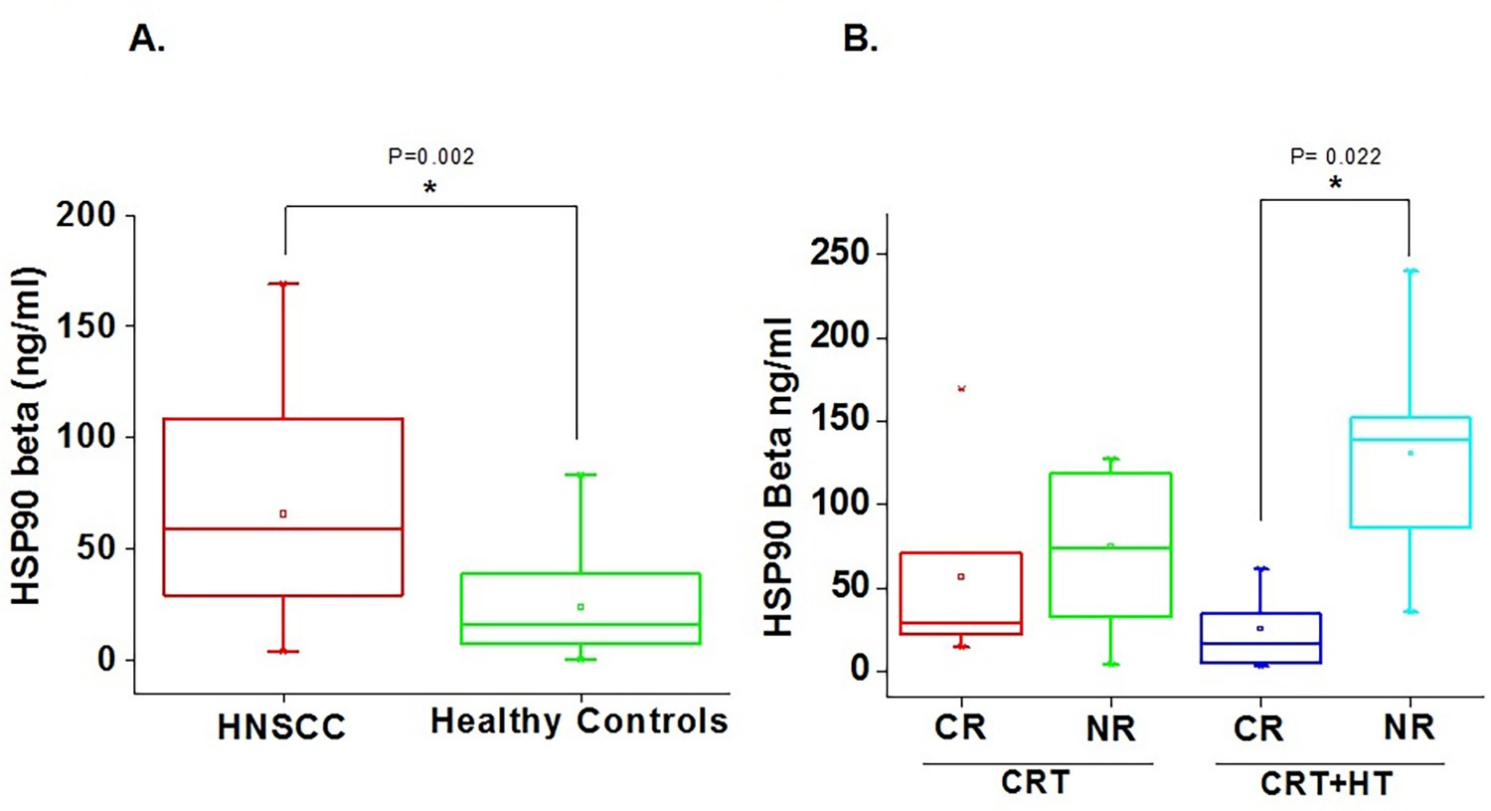
Box-Whisker plot for serum levels of **(A)** HSP90 beta in HNSCC (N = 14) and Healthy Controls (HC) (N = 32). *Significantly different at P<0.05 as determined by Mann-Whitney test. **(B)** HSP90 beta in HNSCC patients subjected to CRT (N = 13) or CRT+HT (N = 11). The serum samples were collected prior to HT (labeled as CRT) and 24 h after HT (labeled as CRT+HT). HNSCC patients have been categorized as complete responders (CR) based on complete response to treatment or with no or partial response or progressive disease or with stable disease as non-responders (NR). *Significantly different at P<0.05 as determined by one way ANNOVA.

To establish the diagnostic potential of HSP90 in clinical scenario, we compared the serum levels of HSP90 alpha and beta in HNSCC (N = 14, Mean age: 59.2±12.7 years) and HC (N = 32, Mean age: 52.2±16.2 years) **(Table 1)**. Results showed significantly higher level of HSP90 beta (2.7 fold) in serum samples of HNSCC (65.6±13.08 ng/ml) compared to HC (23.5±3.8 ng/ml) with a P value of 0.002 **(Fig 1A & Table 2).** However, the levels of HSP90 alpha did not show significant difference between the HNSCC and HC (**S1 Fig**). An anonymized data of the HNSCC patients and healthy controls for HSP90 beta is being mentioned in (**S1 and S2 Tables**).

**Table 1:** Sample demographics.

Parameters	HNSCC patients	Healthy Controls
**No.**	14	32
**Mean Age**	59.2 ± 12.7	52.2 ± 16.2
**Age Range**	38–80 years	32–80 years
**Male**	14 (93.3%)	30 (93.75%)
**Female**	1 (6.7%)	2 (6.25%)

**Table 2:** HSP90 beta values in HNSCC patients (N = 14) and healthy controls (N = 32).

	Concentration of HSP90 beta (ng/ml)	
	HNSCC patients	Healthy controls
**Range**	3.7 to 169.02	0.16 to 83.4
**Median**	58.7	15.6
**Mean**	65.6	23.5
**SEM**	13.08	3.8

### 3.2 Comparison of HSP90 beta levels between complete (CR) and partial/non-responders (NR) in serum samples of HNSCC patients subjected to CRT or CRT+HT

To further determine the correlation between serum levels of HSP90 beta in HNSCC and their response to HT treatment, the HNSCC patients were categorized as CR or NR at the end of chemo-radiation therapy (CRT)+HT treatment session. Baseline serum samples were collected before HT (but after completion of CRT) and compared with the HSP90 beta levels in CRT+HT cohort, at 24 h after completion of HT session. Our results showed in-significant difference in the serum levels of HSP90 beta between CR and NR in the CRT cohort. However, 24 h after HT, significantly lower (~ 5 fold) serum levels of HSP90 beta was observed in CR (25.62±9.04 ng/ml) compared to NR (130.51±34.23 ng/ml) **(Fig 1B and Table 3 & S3 Table)**.

**Table 3:** Multi-variate analysis of CR and NR by one way ANNOVA.

One way ANNOVA	Mean Diff	SEM	t Value	P-Value	95% CI
**NR(CRT) CR(CRT)**	18.30	29.42	0.62	1	-67.8–104.42
**CR(CRT+HT) CR(CRT)**	-30.70	30.53	-1.005	1	-120.07–58.66
**CR(CRT+HT) NR(CRT)**	-49.00	29.42	-1.66	0.66	-135.12–37.11
**NR(CRT+HT) CR(CRT)**	74.18	32.02	2.31	0.18	-19.54–167.92
**NR(CRT+HT) NR(CRT)**	55.88	30.96	1.80	0.51	-34.75–146.52
**NR(CRT+HT) CR(CRT+HT)** *	104.89*	32.02	3.27	**0.022**	11.15–198.62

* Significantly different at P<0.05

CR: Complete responders, NR: Patients with no response or partial response or stable disease or progressive disease, CRT: Chemo-Radiation Therapy, HT: Hyperthermia therapy, Mean Diff: Difference between the Means.

### 3.3 Serum HSP90 beta showed significant efficacy for detection of HNSCC and their clinical response to HT

**Fig 2:**
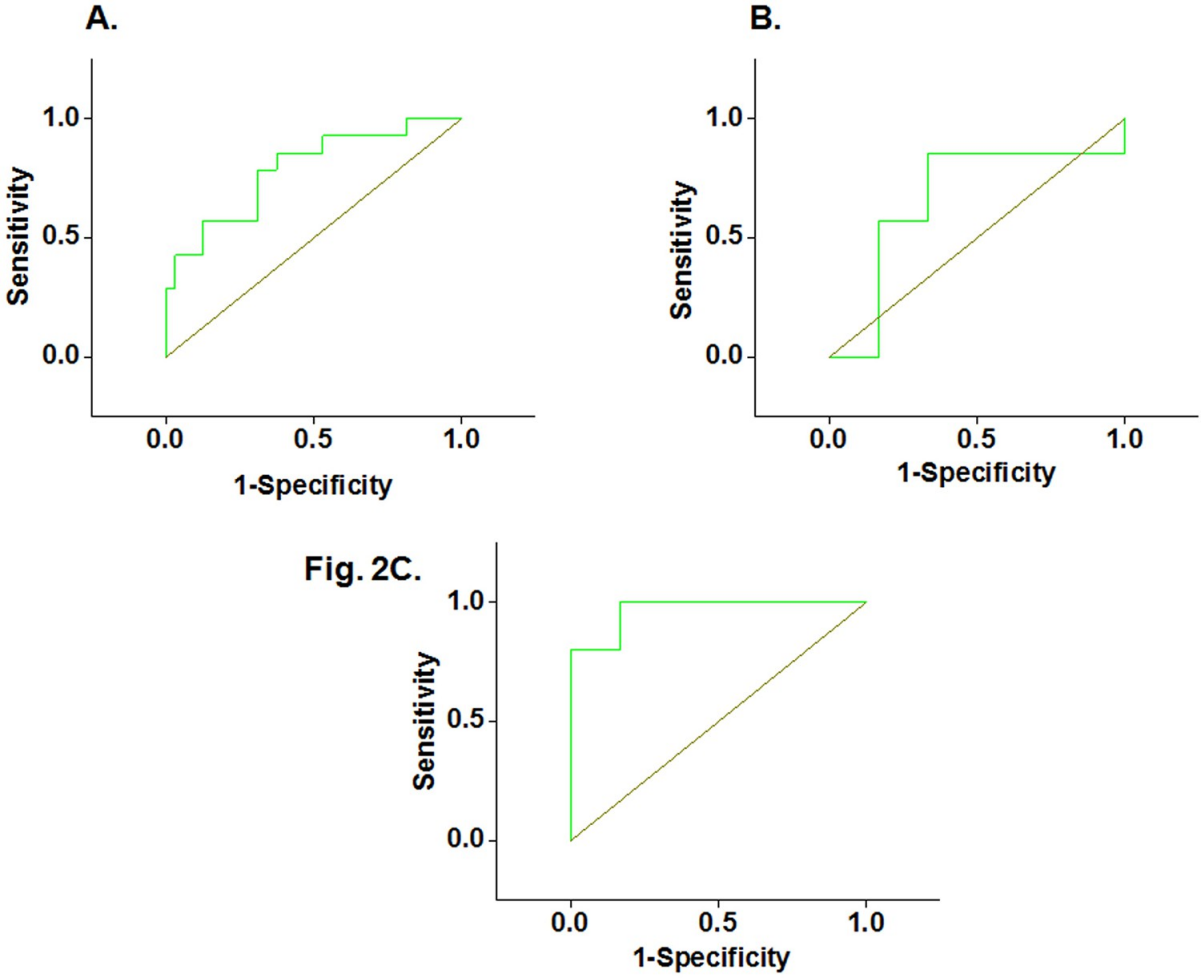
ROC curve analysis for HSP90 beta between **(A)** HNSCC (N = 14) and HC (N = 32) and **(B)** CR (N = 7) and NR (N = 7) for serum samples collected after chemo-radiation therapy (CRT) but before HT and between **(C)** CR (N = 6) and NR (N = 5) for serum samples collected 24 h after HT in CRT+HT cohort.

ROC curve analysis showed an AUC of 0.78 (95% CI: 0.64–0.92) with a sensitivity of 84.6% and specificity of 62.5% at a cut-off value of >22.6 ng/ml for distinguishing HNSCC from HC (P <0.05) **(Fig 2A)**. Nevertheless, no significant difference (P = 0.17) was observed between CR and NR in case of CRT cohort **(Fig 2B & Tables 3 and 4)**. However, in CRT+HT cohort, a significant difference (P<0.05) in the serum levels of HSP90 beta was observed between CR and NR **(Fig 2C & Tables 3 and 4)** with an AUC of 0.96 (95% CI: 0.86–1.06) and a sensitivity and specificity of 100 and 80%, respectively, at a cut-off value of >35.4 ng/ml. Youden’s index was found to be 0.47 and 0.8 for distinguishing HNSCC versus HC and CR versus NR, respectively in serum samples of CRT+HT **(Table 4)**.

**Table 4:** Cut-off values of variables analyzed with respective diagnostic test parameters.

	HNSCC vs HC	CR vs NR (CRT)	CR vs NR (CRT+HT)
**Youden’s index (J)**	0.47	0.45	0.80
**Cut-Off value (ng/ml)**	>22.6	>32.8	>35.4
**Sensitivity**	84.6%	85.7%	100%
**Specificity**	62.5%	60.0%	80%
**Area under curve (AUC)**	0.78	0.66	0.96
**95% CI for AUC**	0.64–0.92	0.33–0.99	0.86–1.06
**P-Value**	P = 0.002	P = 0.17	P = 0.01

HNSCC: Head and Neck squamous cell carcinoma, HC: Healthy Controls, CR: Complete responders, NR: Patients with no response or partial response or stable disease or progressive disease, CRT: Chemo-Radiation Therapy, HT: Hyperthermia therapy, CI: Confidence interval.

## 4. Discussion

Hyperthermia therapy (HT) has emerged as a promising approach for improving the efficacy of CRT in HNSCC [7–9]. Despite of the advancements in the therapeutic strategies, the improvement in overall survival of HNSCC patients has been dismal. Major factors contributing to the poor prognosis of HNSCC patients has been the lack of diagnosis of disease at an early stage and the associated failure of treatment at the advanced stages of the disease [26, 27]. Delayed diagnosis of HNSCC has been associated with poor prognosis with a 5-year median overall survival of 42% at stage IV in Indian patients [27]. With the evolving understanding of the molecular basis of human malignancies, there has been great interest in determining whether serum biomarkers might aid in early diagnosis and guide treatment decision for the cancer patients [28].

Heat shock proteins (HSPs), especially, HSP70, HSP27 and HSP90 have been known to be released in the extra-cellular environment in various solid tumors [29]. Elevated expression of HSP90 has been found to correlate with tumor cell proliferation, tumor stage and poor clinical outcome, suggesting potential use of HSP90 expression in cancer diagnosis and prognosis [18–20]. Correspondingly, there are studies highlighting the role of HSP inhibitors used in conjunction with HT to improve its overall therapeutic efficacy in solid tumors. This effect is conjectured on the ability of the HSP inhibitors to potentiate the cyto-toxic and/or anti-proliferative effects of HT [30]. Concurring with these reports, our previous studies in murine fibrosarcoma tumor models (both *in vitro* and *in vivo*) showed differential expression of HSP90 in cancer cells and tumor lysates. Moreover, the modulation in the levels of HSP90 correlated with the response of tumor to MHT [22]. Therefore, to further evaluate the diagnostic efficacy of HSP90 in clinical settings, we studied the serum levels of HSP90 beta and alpha in HNSCC patients. HNSCC model was specifically chosen owing to the proven advantage of HT in HNSCC for the improvement of therapeutic efficacy of chemo-radiotherapy [7–9].

In the present pilot study, HNSCC patients were found to have significantly higher expression of HSP90 beta but not HSP90 alpha **(S1 Fig)** as compared to HC. This is contrary to other reports demonstrating association of elevated plasma levels of HSP90 alpha with presence of lung [19] or liver [20] cancer, compared to HC. Interestingly, both the isoforms of HSP90, viz., alpha [19, 20] and beta [18] have been implicated as diagnostic serum/plasma biomarkers for several solid tumors. The difference in our observation (insignificant change in HSP90 alpha) compared to these studies may be attributed to the different cancer types/nature of sample. Thus, a correlation between the HSP90 isoforms and their predictive efficacy in a particular tumor type is not well reported and needs further investigation.

Furthermore, our results showed significantly higher levels of HSP90 beta in partial or non-responders (NR) as compared to CR in serum samples collected after 24h of completion of HT in CRT+HT cohort. ROC analysis showed a sensitivity of 84.6 and 100% and specificity of 62.5 and 80% for distinguishing the HC from the HNSCC patients (P-value <0.05) and CR from NR (P<0.05) in CRT+HT cohort, respectively. These values are comparable to another study by Yamashita et al., wherein a sensitivity and specificity of 57.3 and 85.3%, respectively, was observed for predicting HNSCC using serum midkine levels as a bio-marker [31]. Thus, our preliminary results demonstrate the diagnostic efficacy of HSP90 beta for HNSCC and its predictive efficacy for response to HT in HNSCC patients.

## 4. Conclusions

Present pilot study suggests the potential of serum HSP90 beta as a diagnostic serum bio-marker for HNSCC and predicting their response to HT. However, more patients need to be incorporated in the study for improving the statistical power and clinical translation of the research.

## Supporting information

S1 Table Anonymized data for levels of HSP90 beta in serum samples of HNSCC (15 no.) patients subjected to CRT + HT. (DOC)

S2 Table Anonymized data for levels of HSP90 beta in serum samples of Healthy Controls (42 no.). (DOC)

S3 Table Median, Mean and SEM of HSP90 beta in serum samples of HNSCC patients with complete response categorized as complete responders (CR) and with partial or no response or progressive disease or stable disease as non-responders (NR). The HNSCC patients were subjected to CRT and serum samples were analyzed before HT (labeled as CRT) and 24 h after subjecting the same patients to HT (labeled as CRT+HT) for HSP90 beta levels by ELISA.
(DOC)

S1 Fig Box-Whisker plot for serum levels of HSP90 alpha in HNSCC (N = 6) and Healthy Controls (HC) (N = 15). Statistical analysis by Mann-Whitney test suggested no significant difference between HNSCC and Healthy Controls.
(DOC)
