# Supplementary material for: Diagnostic potential of serum HSP90 beta for HNSCC and its therapeutic prognosis after local hyperthermia therapy
Source: PLoS One. 2023 Nov 9;18(11):e0281919. doi: 10.1371/journal.pone.0281919 (PMC10635538; doi:10.1371/journal.pone.0281919)
Supplement: S1 Table — (DOC) [file pone.0281919.s001.doc]

**Supplementary File**

**Supplementary Table T1 : Anonymized data for levels of HSP90 beta in serum samples of HNSCC (N=15) patients subjected to CRT + HT**

|  |  |  |  | **HSP90 beta Levels (ng/ml)** | |  |
| --- | --- | --- | --- | --- | --- | --- |
| **S.No.** | **Patient ID** | **Age** | **Gender** | **CRT** | **CRT+ HT** | **Response** |
| **1** | **P1** | **54** | **M** | **ND** | **5.30** | **CR** |
| **2** | **P2** | **41** | **M** | **108.69** | **138.40** | **NR** |
| **3** | **P3** | **65** | **F** | **22.65** | **2.78** | **CR** |
| **4** | **P4** | **62** | **M** | **14.13** | **34.12** | **CR** |
| **5** | **P5** | **64** | **M** | **126.88** | **152.47** |  |
| **6** | **P6** | **45** | **M** | **58.08** | **86.59** | **SD** |
| **7** | **P7** | **74** | **M** | **70.80** | **33.72** | **CR** |
| **8** | **P8** | **58** | **M** | **32.39** | **16.22** | **CR** |
| **9** | **P9** | **38** | **M** | **32.83** | **35.41** | **PR** |
| **10** | **P10** | **80** | **M** | **118.82** | **239.69** | **NR** |
| **11** | **P11** | **58** | **M** | **73.36** |  | **NR** |
| **12** | **P12** | **60** | **M** | **169.02** |  | **CR** |
| **13** | **P13** | **70** | **M** |  | **61.59** | **CR** |
| **14** | **P14** | **74** | **M** | **28.96** |  | **CR** |
| **15** | **P15** | **45** | **M** | **3.73** |  | **PR** |

CR: Complete responders, NR: Patients with no response, PR: Partial response, SD: Stable disease or PD: progressive disease, CRT: Chemo-Radiation Therapy, HT: Hyperthermia therapy

ND : Non-Detectable (Below the Minimum Detectable Dose for the Kit)
