# Supplementary material for: Diagnostic potential of serum HSP90 beta for HNSCC and its therapeutic prognosis after local hyperthermia therapy
Source: PLoS One. 2023 Nov 9;18(11):e0281919. doi: 10.1371/journal.pone.0281919 (PMC10635538; doi:10.1371/journal.pone.0281919)
Supplement: S2 Table — (DOC) [file pone.0281919.s002.doc]

**Supplementary File**

**Supplementary Table T2 : Anonymized data for levels of HSP90 beta in serum samples of Healthy Controls (N=42**)

| **S. No.** | **Healthy Control ID** | **HSP90 beta levels (ng/ml)** |
| --- | --- | --- |
| **1.** | **HC1** | **11.93** |
| **2.** | **HC2** | **8.69** |
| **3.** | **HC3** | **24.30** |
| **4.** | **HC4** | **13.57** |
| **5.** | **HC5** | **60.20** |
| **6.** | **HC6** | **83.42** |
| **7.** | **HC7** | **14.37** |
| **8.** | **HC8** | **9.09** |
| **9.** | **HC9** | **63.70** |
| **10.** | **HC10** | **4.95** |
| **11.** | **HC11** | **37.92** |
| **12.** | **HC12** | **26.24** |
| **13.** | **HC13** | **13.32** |
| **14.** | **HC14** | **63.32** |
| **15.** | **HC15** | **34.75** |
| **16.** | **HC16** | **ND** |
| **17.** | **HC17** | **ND** |
| **18.** | **HC18** | **ND** |
| **19.** | **HC19** | **ND** |
| **20.** | **HC20** | **3.32** |
| **21.** | **HC21** | **2.49** |
| **22.** | **HC22** | **3.68** |
| **23.** | **HC23** | **0.59** |
| **24.** | **HC24** | **0.16** |
| **25.** | **HC25** | **ND** |
| **26.** | **HC26** | **10.95** |
| **27.** | **HC27** | **18.89** |
| **28.** | **HC28** | **50.24** |
| **29.** | **HC29** | **1.80** |
| **30.** | **HC30** | **5.86** |
| **31.** | **HC31** | **ND** |
| **32.** | **HC32** | **ND** |
| **33.** | **HC33** | **ND** |
| **34.** | **HC34** | **ND** |
| **35.** | **HC35** | **41.70** |
| **36.** | **HC36** | **8.47** |
| **37.** | **HC37** | **ND** |
| **38.** | **HC38** | **17.007** |
| **39.** | **HC39** | **41.94** |
| **40.** | **HC40** | **20.77** |
| **41.** | **HC41** | **17.33** |
| **42.** | **HC42** | **39.23** |
