# Supplementary material for: Diagnostic potential of serum HSP90 beta for HNSCC and its therapeutic prognosis after local hyperthermia therapy
Source: PLoS One. 2023 Nov 9;18(11):e0281919. doi: 10.1371/journal.pone.0281919 (PMC10635538; doi:10.1371/journal.pone.0281919)
Supplement: S3 Table — The HNSCC patients were subjected to CRT and serum samples were analyzed before HT (labeled as CRT) and 24 h after subjecting the same patients to HT (labeled as CRT+HT) for HSP90 beta levels by ELISA. (DOC) [file pone.0281919.s003.doc]

**Supplementary File**

**Supplementary Table T3** : Median, Mean and SEM of HSP90 beta in serum samples of HNSCC patients with complete response categorized as complete responders (CR) and with partial or no response or progressive disease or stable disease as non-responders (NR). The HNSCC patients were subjected to CRT and serum samples were analyzed before HT (labeled as CRT) and 24 h after subjecting the same patients to HT (labeled as CRT+HT) for HSP90 beta levels by ELISA.

|  | **Median HSP90 beta (ng/ml)** | **Mean HSP90 beta (ng/ml)** | **SEM** | **Range (ng/ml)** |
| --- | --- | --- | --- | --- |
| **CR (CRT)** | 30.6 | 56.3 | 23.9 | 14.1 to 169.02 |
| **NR (CRT)** | 73.3 | 74.6 | 17.5 | 3.7 to 126.8 |
| **CR (CRT+HT)** | 24.9 | 25.6 | 9.04 | 2.7 to 61.5 |
| **NR (CRT+HT)** | 138.4 | 130.5 | 34.2 | 35.4 to 239.6 |
