# Supplementary material for: Diagnostic potential of serum HSP90 beta for HNSCC and its therapeutic prognosis after local hyperthermia therapy
Source: PLoS One. 2023 Nov 9;18(11):e0281919. doi: 10.1371/journal.pone.0281919 (PMC10635538; doi:10.1371/journal.pone.0281919)
Supplement: S1 Fig — Statistical analysis by Mann-Whitney test suggested no significant difference between HNSCC and Healthy Controls. (DOC) [file pone.0281919.s004.doc]

**Supplementary File**

**Supplementary Figure:**

**Fig. S1**

**P=0.38**

**Healthy Controls**

**HNSCC**

**Fig. S1.** Box-Whisker plot for serum levels of HSP90 alpha in HNSCC (N=6) and Healthy Controls (HC) (N=15). Statistical analysis by Mann-Whitney test suggested no significant difference between HNSCC and Healthy Controls.
